# A millimeter-wave automotive radar with high angular resolution for identification of closely spaced on-road obstacles

**DOI:** 10.1038/s41598-023-30406-4

**Published:** 2023-02-24

**Authors:** Ran Sun, Kouhei Suzuki, Yuri Owada, Shigeki Takeda, Masahiro Umehira, Xiaoyan Wang, Hiroshi Kuroda

**Affiliations:** 1 College of Engineering, Ibaraki University, Ibaraki, 316-8511 Japan; 2 Hitachi Astemo, Ltd., Ibaraki, 312-8503 Japan

**Keywords:** Electrical and electronic engineering, Applied physics

## Abstract

Frequency-modulated continuous wave radar techniques typically have inadequate angular resolutions due to the limited aperture sizes of antenna arrays in spite of employing multiple-input multiple-output (MIMO) techniques. Therefore, despite the existence of multiple objects, angularly close objects with similar distances and relative velocities are recognized as one single object. Autonomous driving requires the accurate recognition of road conditions. This requirement is one of the critical issues to be solved to distinguish significantly close objects. This paper proposes a technique referred to as an antenna element space pseudo-peak suppressing (APPS) method to resolve angularly close targets. The proposed APPS method aims to identify closely spaced objects on roads. These angularly close targets cause a single peak in a spatial spectrum obtained by a beamformer-based angle estimation. The APPS considers this single peak as pseudo. The APPS radar cancels this pseudo peak from the spatial spectrum. Then, the obtained residual received signal is analyzed. With these procedures, the APPS identifies the number of targets. The APPS also estimates the target angles. The proposed APPS is experimentally validated using a typical single-chip MIMO-based radar evaluation board with three transmit (TX) and four receive (RX) antennas. The experimental results confirm that the proposed APPS successfully resolves angularly close pseudo targets with an angle difference of approximately $0.5^\circ$.

## Introduction

Because of increasing demand and interest in advanced driver assistance systems (ADASs) and autonomous driving systems, millimeter-wave radars are attracting much attention^1,2^. Compared with LiDAR (light detection and ranging), cameras and optical sensors, the cost of millimeter-wave radar is low, since it only consists of an integrated circuit (IC) and printed antennas. Moreover, compared to traditional camera imaging systems, millimeter-wave radars are more effective in bad weather conditions (fog and rain) and nonline of sight (NLOS) target detection (curves on roads)^3,4^. Frequency-modulated continuous wave (FMCW) and fast chirp modulation (FCM) radars can be used to inherently measure ranges and relative velocities between a radar and targets^5,6^. In addition, these radars can be used to measure target angles if equipped with arrayed antennas and multiple-input multiple-output (MIMO) radar functions^7^. The abovementioned angle estimation ability is required to resolve multiple targets when these targets have almost the same ranges and relative velocities. In particular, the detection and characterization of multiple targets on a road will play significantly important roles in ADAS and autonomous driving applications.

To equip various kinds of vehicles with millimeter-wave radars, including cars, tricycle, scooters, and bicycles, their production costs need to be low, and the form factors should be small. Furthermore, four-dimensional (4D) radar measuring ranges, relative velocities, azimuthal and elevation angles have been actively developed worldwide^8,9^. In these 4D radar systems, angularly close targets need to be individually resolved because the radars need to identify all the possible objects and obstacles on roads. To recognize targets, various kinds of higher resolution millimeter-wave radars have been studied. A beam switching method was introduced in^10^, a radar imaging and neural network were used in^11^ to characterize road surfaces, and wide aperture and cascading radar units were employed in^12,13^ and^14,15^, respectively. Leak mitigation has been considered in^16^. Human recognition has been investigated in^17^ and^18^. A calibration method and an indoor radar imaging system to suppress the influence of surrounding clutter have been shown in^19^ and^20^. However, all of these studies require large-scale hardware and complicated signal processing. For example,^10^ need additional circuitries for transmit antenna beam switching.^12–14^ and^15^ are much larger than a 3T_X_–4R_X_ MIMO radar with a single radar IC.^11,17^ and^18^ use high computational complexity artificial intelligence (AI) algorithms.^21^ also uses a high computational complexity AI algorithm: convolutional neural network (CNN) for multi-target classification. Moreover, some classical radar signal processing techniques such as virtual antennas^22^, and multiple signal classification (MUSIC) algorithms^23^, are also adopted to the FMCW radar. However, beamformer-based angle estimations are typically used in practical automotive millimeter radars^5^, where these angle estimation methods are sometimes referred to as Angle Fast Fourier Transform (FFT). Maximum likelihood estimation is also a classic radar signal processing technique, but it has high computational complexity and cannot resolve extremely close targets^24^. Therefore, radar with low-complexity signal processing and small form factors is urgently needed.

To this end, this paper proposes a higher angular resolution technique by introducing an antenna element space pseudo peak suppressing method, which has extremely low signal processing cost and just use 3T_X_–4R_X_ MIMO radar with a single radar IC^25^. The proposed method is based on an antenna element space interference canceling (AIC) technique that was proposed in^26^ for automotive millimeter wave FMCW radars. The original proposed AIC technique has been previously introduced into multiple target radar imaging for suppressing the influence of side-lobes from large targets. We extend this method and refer to it as antenna element space pseudo peak suppression (APPS) in this paper. RELAX and CLEAN algorithms^27–29^ first introduced the signal canceling concept to array antenna processing. On the other hand, the proposed APPS for automotive millimeter-wave FMCW radars focuses on realizing the detection of significantly angularly close targets and higher angular resolution performance. These requirements are recent urgent problems to be solved in automotive FMCW radars. If two targets are significantly angularly close, an ordinary beamformer method observes a single peak in a spatial spectrum because of the inadequate aperture of the antenna array. The APPS processing considers this single peak as pseudo and cancels this pseudo peak in the antenna element space of the antenna array. The beamforming-based angle estimation is applied for the resulting input signal vector again to analyze the residual received signal component. With this procedure, the APPS radar system identifies the number of targets by the residual signal after canceling the pseudo peak. Furthermore, the APPS also estimates the angles of the targets. The proposed APPS is experimentally validated using a typical single-chip radar evaluation board, where a multiple-input multiple-output radar architecture is equipped with three transmit (T_X_) and four receive (R_X_) antennas. The experimental results confirm that the proposed APPS can successfully resolve ground-placed targets that have an angle difference of only $0.5^\circ$.

**Figure 1:**
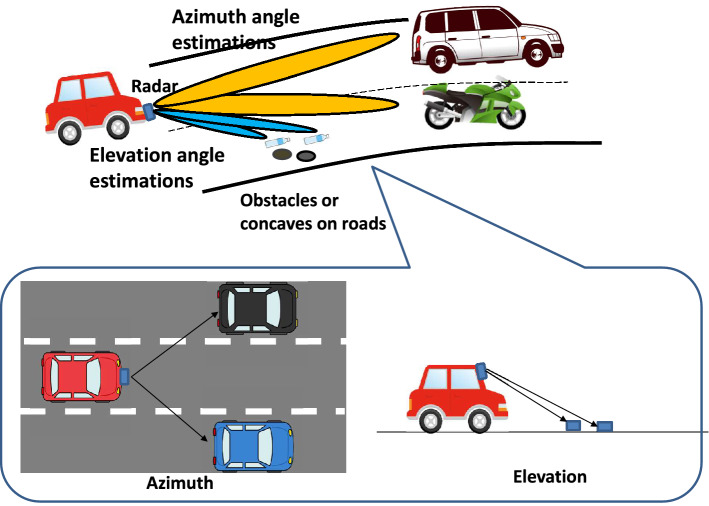
Need for higher resolution angle estimations.

APPS is developed to resolve angularly extremely close targets, as shown in Fig. 1. Radars resolve targets in the azimuth or elevation planes. To this end, horizontally, vertically or both arranged array antennas are used in conjunction with MIMO radar techniques. FMCW radars can distinguish between multiple targets in terms of range and relative velocity. However, if these targets have almost the same range and velocity, angle estimations are necessary to individually locate these targets. The bottom left figure shown in Fig. 1 illustrates the scenario in which the front targets have the same range and velocity, and the bottom right figure illustrates the scenario in which the targets on a road have a spacing less than the range resolution of a FMCW radar. The problem is that the number of targets and their angles cannot be correctly detected due to the limited angular or range resolution of a FMCW radar.

## Methods

As previously stated, because FMCW radars can distinguish between multiple targets in terms of range and relative velocity, most targets can be resolved based on these inherent features of FMCW radars. Hence, the number of targets that need to be resolved by angle estimation only occurs when multiple targets have the same distance and velocity, and, thus, is very rare in reality. Therefore, the number of targets that require angle estimation is assumed to be at most two in this paper. An ordinary beamforming method cannot be used to distinguish targets when they are angularly close to each other. In this case, a pseudo peak appears in the spatial spectrum despite the presence of two targets. To overcome this problem, APPS is introduced to FMCW radar systems.

**Figure 2:**
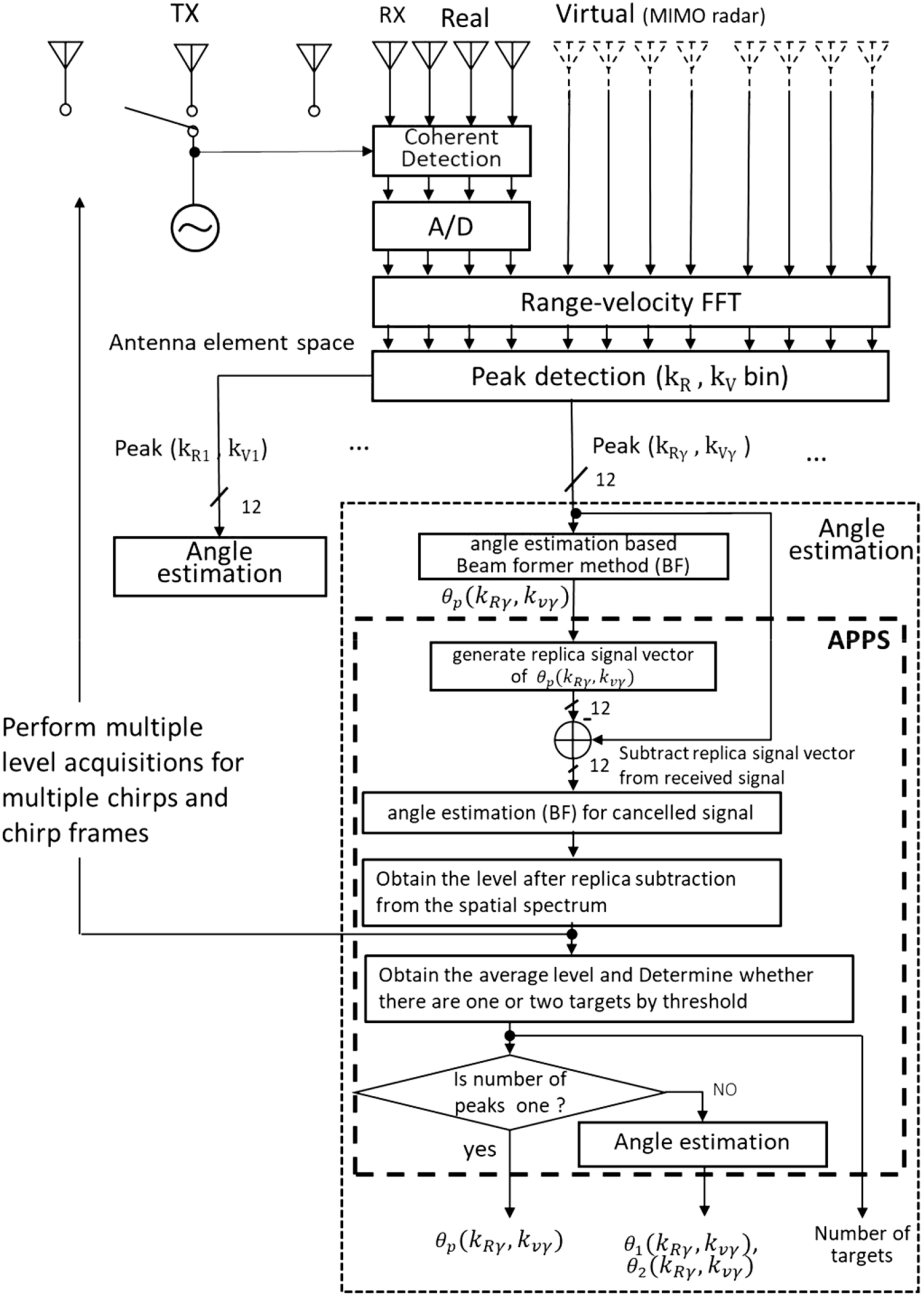
System structure of the proposed APPS-radar.

APPS processing is based on the antenna element space interference canceling radar^26^. AIC radar processing enables the provision of a replica received signal vector for the maximum peak angle of a spatial spectrum, which is generated by beamforming-based angle estimation.

**Figure 3:**
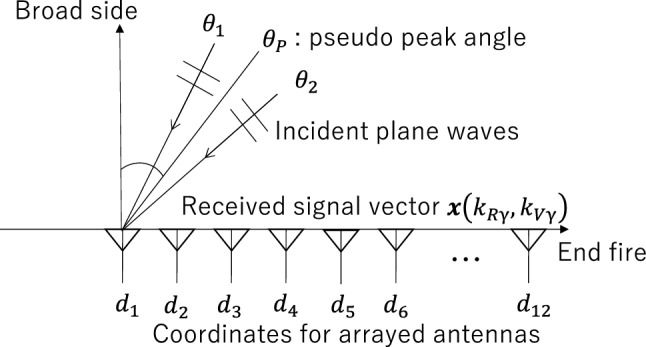
Definitions for the coordinates, incident waves and the antenna arrangement.

Figure 2 shows a block diagram of the radar with APPS. This block diagram assumes that a typical single-chip radar integrated circuit (IC) has three TX and four RX radio frequency (RF) circuits. The radar transmits radio waves, and then array antennas receive the reflected waves from the targets. The use of a MIMO radar architecture is assumed here. The received signals can be virtually enhanced to a receive array antenna with 12 antenna elements. Two-dimensional FFTs are applied to obtain range and velocity bins. This processing enables radars to resolve multiple targets based on differences in range and velocity. To locate different targets, the beamforming-based angle estimation algorithm is applied to each peak. A received signal vector for one of the range and velocity peaks is given by the following equation. Figure 3 shows the definitions for the coordinates, incident waves, and an antenna arrangement. The received signal vector for the $\gamma$-th peak can be defined as

1 $$\begin{aligned} {\varvec{x}}(k_{R\gamma },k_{V\gamma }) = h_1 {\varvec{a}}(\theta _1) + h_2 {\varvec{a}}(\theta _2)+ {\varvec{n}}, \end{aligned}$$

where $k_{R\gamma }$ and $k_{V\gamma }$ are indices for the range and velocity bins, respectively, and $\theta _1$and $\theta _2$ are incident angles for plane waves, ${\varvec{x}}(k_{R \gamma },k_{V \gamma }) = [x_1, x_2, \cdots , x_N]^T,$ *N* is the number of receive antennas, and ${\varvec{n}}$ represents the noise components. For simplicity, the indices, $k_{R\gamma }$ and $k_{V\gamma }$, are omitted except for the received signal vector ${\varvec{x}}\left( k_{R\gamma },k_{V\gamma }\right)$. The noise vector consists of thermal noise and phase/amplitude calibration errors for the RF circuits and antennas. $h_1$ and $h_2$ are complex coefficients of the two incident waves, and it is assumed that $\left| h_1\right| \approx \left| h_2\right|$. ${\varvec{a}}\left( \theta \right)$ is a steering vector for an incident plane wave at an arbitrary angle.

APPS is applied to each peak. A replica signal is generated for the maximum peak angle based on AIC radar processing, and then this replica is subtracted from the received signal vector. A coefficient for an incident plane wave for the pseudo peak, $h_p$, can be estimated by^26^

2 $$\begin{aligned} h_p=\frac{1}{N}{\varvec{a}}\left( \theta _p\right) ^H{\varvec{x}}\left( k_{R\gamma },k_{V\gamma }\right) , \end{aligned}$$

where $\theta _p$ is an estimated angle for the pseudo peak and $\left( \cdot \right) ^H$ represents the conjugate transpose of a complex vector. Thus, the signal vector after subtracting the replica, ${\varvec{\hat{x}}}\left( k_{R\gamma },k_{V\gamma }\right)$, is given by

3 $$\begin{aligned} \begin{aligned} {\varvec{\hat{x}}}\left( k_{R\gamma },k_{V\gamma }\right)&={\varvec{x}}\left( k_{R\gamma },k_{V\gamma }\right) -h_p{\varvec{a}}\left( \theta _p\right) \\&=\left\{ h_1{\varvec{a}}\left( \theta _1\right) +h_2{\varvec{a}}\left( \theta _2\right) +{\varvec{n}}\right\} -h_p{\varvec{a}}\left( \theta _p\right) . \end{aligned} \end{aligned}$$

The beamforming algorithm is subsequently applied to the resulting signal vector, and then the spatial spectrum obtained for the field of view angles $\theta$ ranging from $-\frac{\pi }{2}$ to $\frac{\pi }{2}\ rad$ is proportional to the power given by

4 $$\begin{aligned} \begin{aligned} P\left( \theta \right)&=\left| {\varvec{a}}^H\left( \theta \right) \hat{{\varvec{x}}}\left( {\varvec{k}}\right) \right| ^2\\&=\left| {\varvec{a}}^H\left( \theta \right) \left[ \left\{ h_1{\varvec{a}}\left( \theta _1\right) +h_2{\varvec{a}}\left( \theta _2\right) +{\varvec{n}}\right\} -h_p{\varvec{a}}\left( \theta _p\right) \right] \right| ^2. \end{aligned} \end{aligned}$$

**Figure 4:**
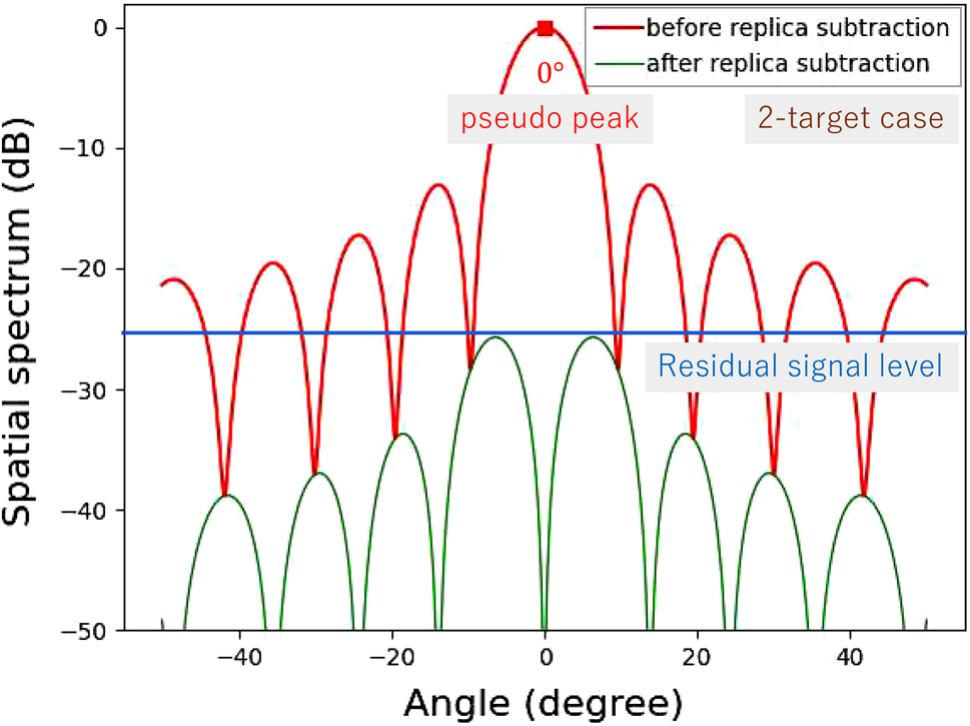
Computationally obtained spatial spectra in the presence of two angularly close targets.

**Figure 5:**
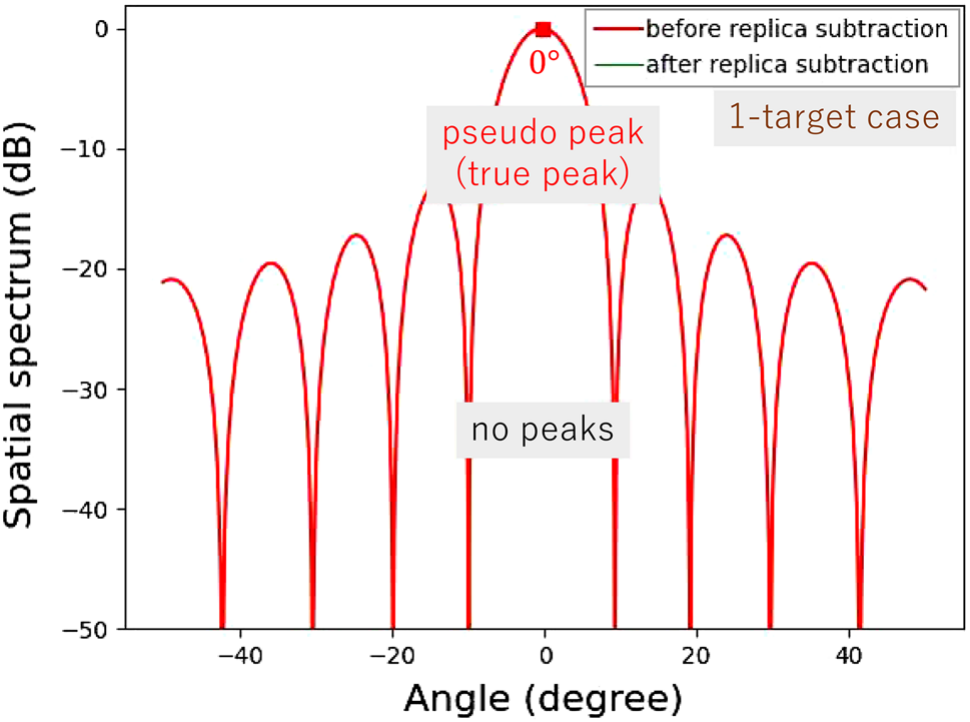
Computationally obtained spatial spectra for a single target.

Figures 4 and 5 show the computationally obtained spatial spectra for two angularly close targets in $\pm 0.30^\circ$ and one target in $0^\circ$, respectively. The phase difference between the two targets in Fig. 4 is set to 90$^{\circ }$. For the two targets shown in Fig. 4, a null point appears in the spatial spectrum, and its angle is $\theta _p$. Hence, a pseudo peak is successfully suppressed. Because the pseudo peak direction $\theta _p$ is different from the true incident angles, $\theta _1$ and $\theta _2$, these incident signal components remain uncancelled at certain levels in the spatial spectrum. These levels are determined by a null pattern for an array antenna. As the incident angle differences decrease, the residual incident wave levels decrease. In contrast, when the number of incident waves is one, the replica signal perfectly cancels the true incident wave. Hence, after subtracting a replica, the incident signal vector ideally becomes a zero vector. Therefore, the spatial spectrum after replica subtraction becomes zero, as shown in Fig. 5.

**Figure 6:**
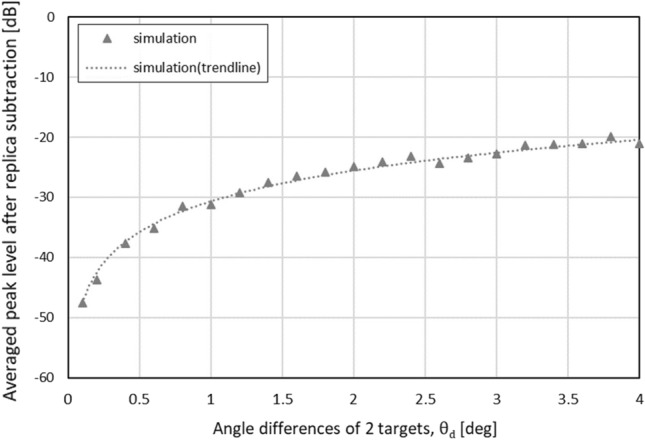
Computationally obtained relationship between average residual levels and angle differences.

The proposed angle estimation mechanism can be explained as follows: If the number of targets is one, the pseudo peak angle corresponds to the estimated angle. However, for two targets, the angles cannot be directly estimated. Therefore, we first obtain the relationship between the average residual levels for the spatial spectra and angle differences by computer simulation, as shown in Fig. 6. These average residual levels are obtained by 300 realizations for each angle difference, where phase differences between two incident waves were determined by using uniformly distributed random variables ranging from 0 to $2\pi \ rad$ for the 300 realizations. The resulting average residual levels for the spatial spectra exponentially increase with increasing angle differences. These average residual levels are normalized by the pseudo peak level. These characteristics are exploited to estimate the true angles of the two targets, $\theta _1$ and $\theta _2$, as $\theta _p-\frac{1}{2}\theta _d$ and $\theta _p+\frac{1}{2}\theta _d$, where $\theta _{d}$ is the estimated angle difference based on Fig. 6.

**Figure 7:**
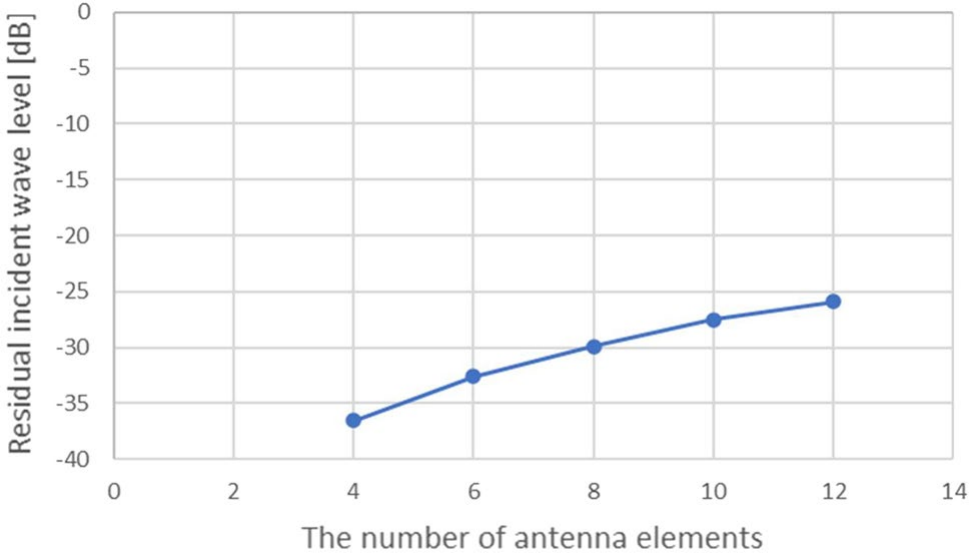
Computationally obtained relationship between number of antenna elements and residual incident wave level.

Moreover, an antenna array with a smaller number of antenna elements widens a null width in a spatial spectrum, reducing the residual incident wave level. The obtained residual incident wave level must be higher than background noise to identify the presence of incident waves in APPS processing. Therefore, the number of antenna elements impacts APPS processing. Figure 7 shows the relationship between the number of antenna elements and a residual incident wave level. This suggests that a higher number of antenna elements leads to a higher residual incident wave level.

**Figure 8:**
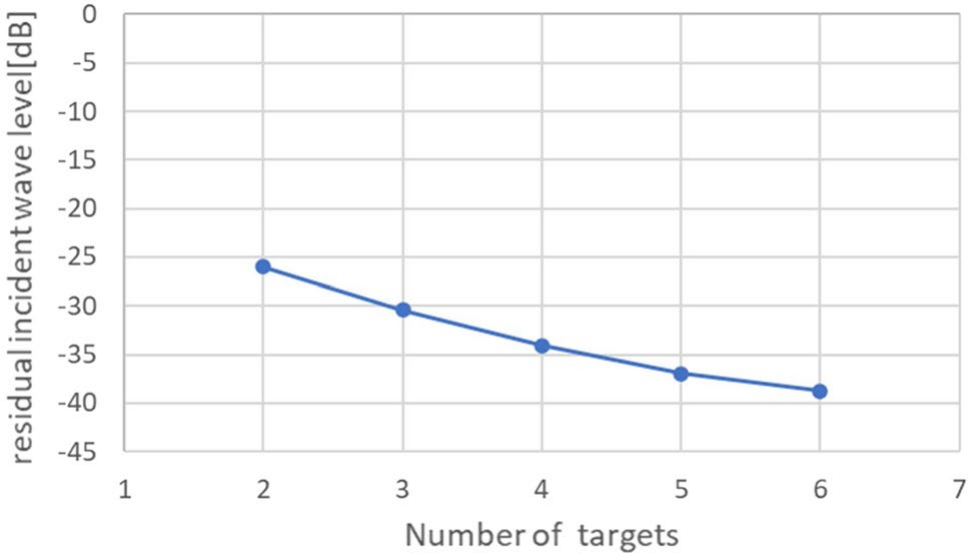
Computationally obtained relationship between number of targets and residual incident wave level.

Figure 8 shows the relationship between the number of targets and a residual incident wave level, which is also obtained by computer simulation. The targets are set at random angles from $-0.30^\circ$ to $+0.30^\circ$, respectively. We can see that the residual incident wave level decreases, as the number of targets increases. It has the potential to use the residual incident wave level for estimating the number of targets. However, the exact estimation of the number of targets is still difficult due to the noise in the practical environment. In this paper, the experiments are limited to no more than two targets.

Based on these characteristics, if a residual component is observed in a spatial spectrum after replica subtraction, the presence of two incident waves is identified. In addition, because an average residual incident wave level is proportional to the difference in incident angle, this characteristic makes it possible to estimate the incident angle. The APPS scheme after obtaining a first spatial spectrum can be summarized as follows:

1. Estimate a pseudo peak angle $\theta _p$,
2. Create a replica signal vector for $\theta _p$, and subtract it from the original receive signal vector,
3. Apply the beamformer method for the received signal vector after subtracting the replica and obtain a spatial spectrum again,
4. Assess the residual levels for the incident waves,
5. Repeat (1) to (4) for multiple chirps or chirp frames to obtain an average residual signal level for the incident waves,
6. Compare the obtained average residual signal level with a threshold determined based on computer simulations or experimental realizations, and estimate the number of incident waves: one or two,
7. Let the incident angle be $\theta _p$ if the number of incident waves is estimated to be one; otherwise, estimate the incident angles $\theta _1$ and $\theta _2$ according to the relationship between incident angles and average residual signal levels shown in Fig.6.

## Results and discussion

**Figure 9:**
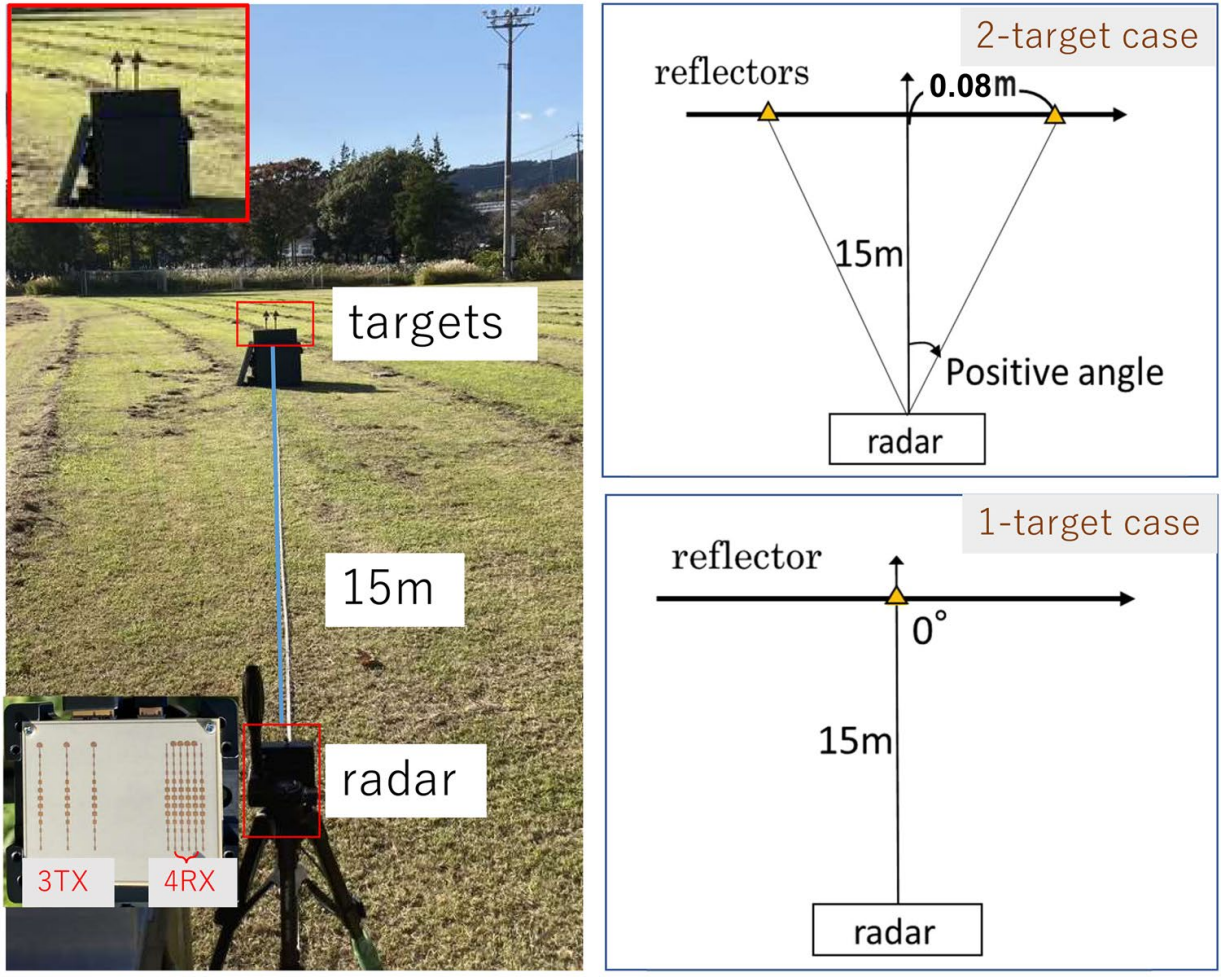
Experimental environment for horizontal angle estimation.

A 79 GHz band MIMO radar-based FMCW/FCM millimeter-wave radar evaluation board, T14 produced by S-TAKAYA, was used in our experiments. This radar is equipped with three TX and four RX RF circuits and antennas, enabling a universal linear array antenna with an antenna element spacing that avoids the generation of grating lobes. The radar offered a 3 TX-4 RX MIMO function. This radar works as a receive antenna array with 12 elements. An inset in Fig. 9 shows the photograph of the antenna structure. Two-dimensional FFTs were employed to obtain range and velocity bins. The number of samples per chirp was 256. Applying 256-point FFT to a chirp in a frame provided the range bins. 32-point FFT for the 32 chirps observed in a frame gave the velocity bins. The range resolution was 0.24 m, where the slope and the sampling rate were 29.92 MHz/$\mu$s and 12.46 Msps, respectively. The radar was vertically installed with a rotation angle of $90^{\circ }$ on a tripod to perform angle estimations in the elevation plane. This experimental setup was used to measure the angles of two targets on the ground. To make a comparison, we also conducted experiments for the detection of horizontal arranged targets using the normal horizontal radar installation.

### Performance analysis for detection of horizontal arranged targets

**Figure 10:**
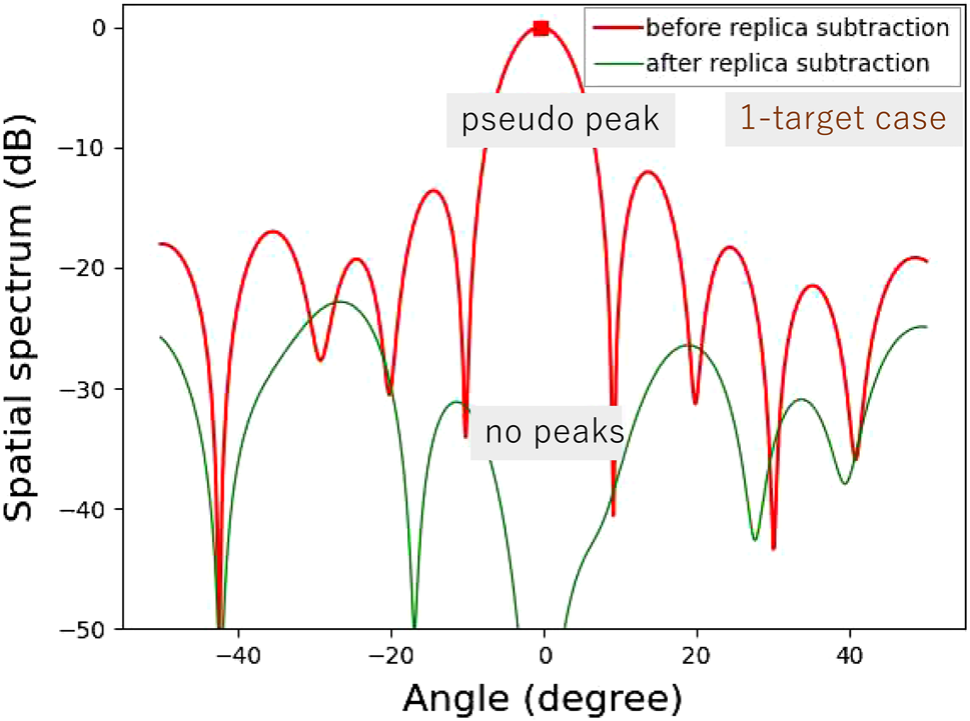
Experimental results for horizontal angle estimation for a single-target case.

**Figure 11:**
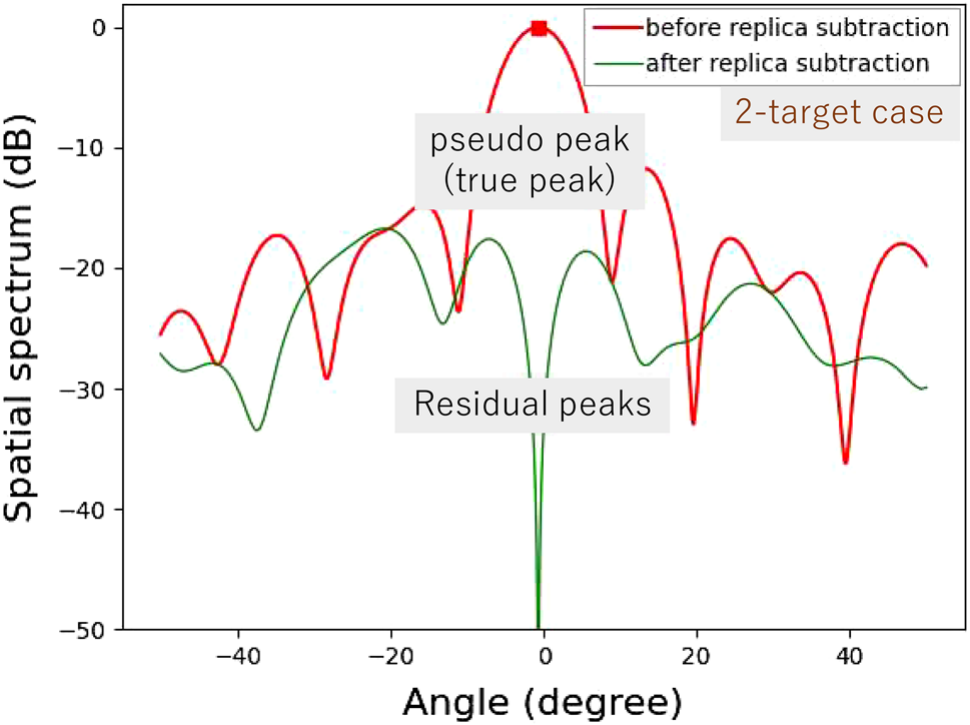
Experimental results for horizontal angle estimation for a two-target case.

Figure 9 shows the experimental environment of horizontal angle estimation. Two triangular pyramid reflectors are used as targets. The radar cross-sectional areas of the targets are 0 dBsm. Two cases are investigated: “single-target case” and “two-target case”. In the single-target case, the target is set at $0^\circ$, as the simulation presented in Figs. 4 and 5. In the two-target case, the horizontal distance between two targets is set to be 0.16 m. The distance between the radar and the center of the two targets is 15 m. Angles of the targets are $\pm {0.31}^\circ$, same to the simulation shown in Section III.

Figures 10 and 11 show experimental results for the single-target case and the two-target case, respectively. For the single target case, the levels around the incident angle are significantly reduced in the spatial spectrum after APPS because the peak shown in the first spatial spectrum was the true peak. Note that the residual spatial spectrum levels outside of the incident angle region are relatively higher because of remaining calibration errors for the TX and RX arrayed antennas and RF circuits. On the other hand, for the two target case, only one pseudo peak appeared in spite of the presence of two targets because the angle difference between the two incident waves is significantly small and beyond the angular resolution of the 12 elements uniform linear array antenna. However, in the spatial spectrum after APPS, because the canceled peak was a pseudo peak, higher spatial spectrum levels due to the residual incident wave components were observed in comparison with that in the single target case. Hence the presence of the two targets were successfully realized.

**Figure 12:**
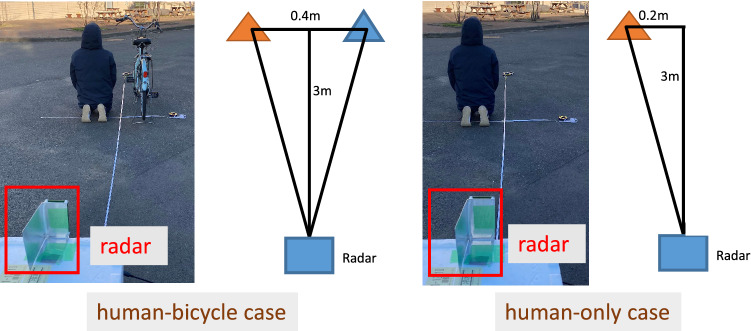
Demonstration environment for horizontal angle estimations of on-road obstacles: human and bicycle.

**Figure 13:**
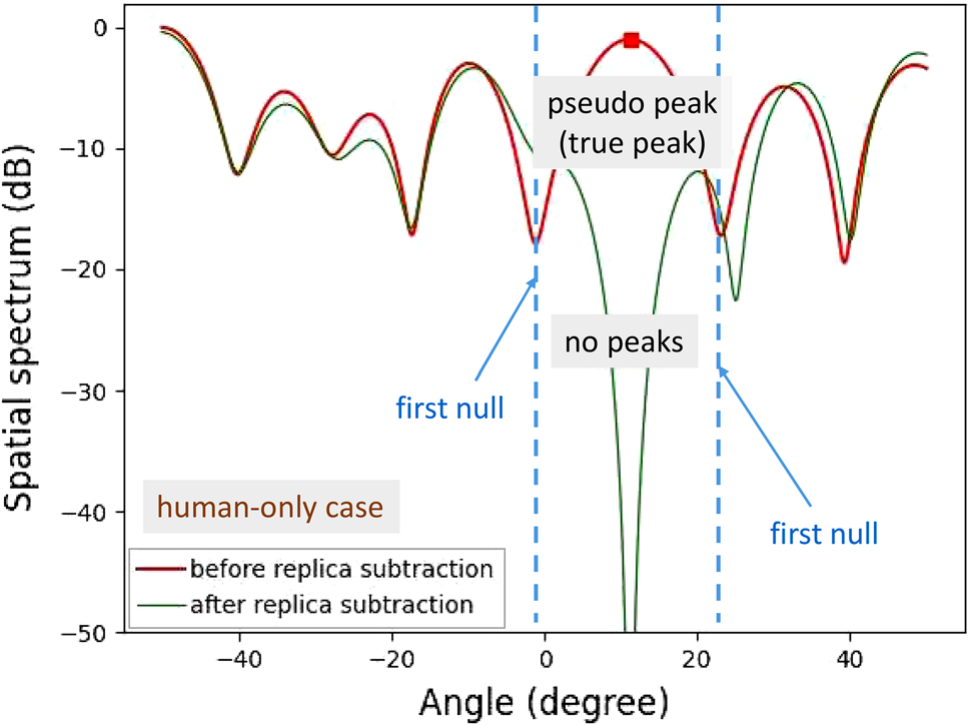
Demonstration results for horizontal angle estimations of on-road obstacles: human-only.

**Figure 14:**
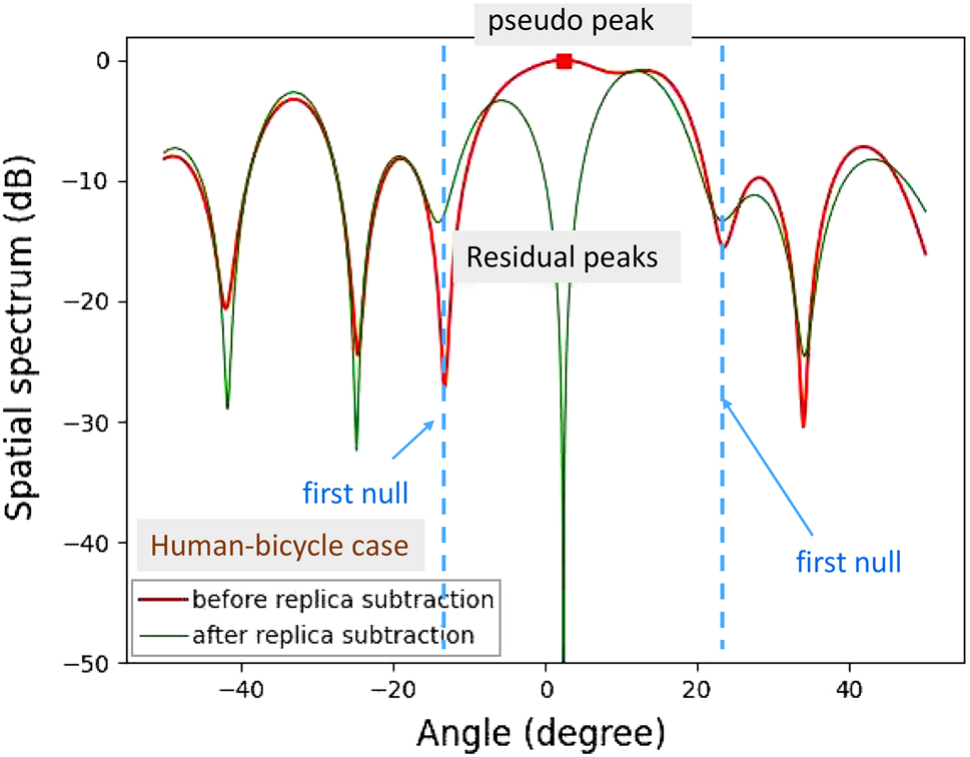
Demonstration results for horizontal angle estimations of on-road obstacles: human-bicycle.

In addition, several experiments for detecting human and bicycle on an asphalt roadway were conducted. Figure 12 depicts the demonstration environment for vertical angle estimations of human and bicycle. Figures 13 and 14 show the results of angle estimations for the human-only case and human-bicycle case, respectively. The residual peaks level for the human-bicycle case in Fig. 14 is obviously higher than that for the human-only case in Fig. 13. These results verify that the proposed method is also effective for actual pedestrians and vehicles.

### Performance analysis for detection of vertically arranged targets

**Figure 15:**
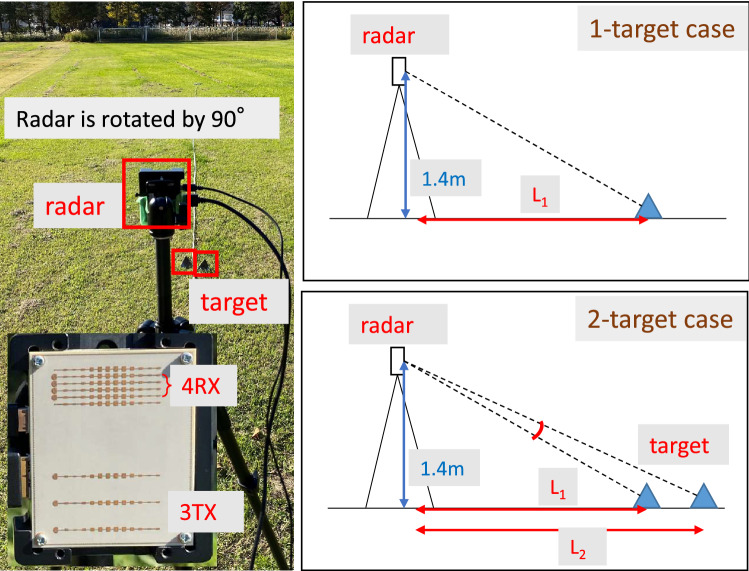
Experimental environment for vertical angle estimations.

Figure 15 shows the experimental environment for angle estimations in the elevation plane. Two identical triangular pyramidal reflectors were placed on the ground, which represent obstacles on a road. Their angles were set to $-23.96^{\circ }$ and $-25.01^{\circ }$. The radar cross-sectional areas of the targets are both 10 dBsm. The height of the radar was 1.4 m. The experiments investigated two cases: “single-target case” and “two-target case”. For the single-target case, the triangular pyramidal reflector was placed 3 m away from the radar. For the two-target cases, the ground projected distances ($L_1, L_2$) between the radar and two targets were 3 m and 3.15 m, respectively.

**Figure 16:**
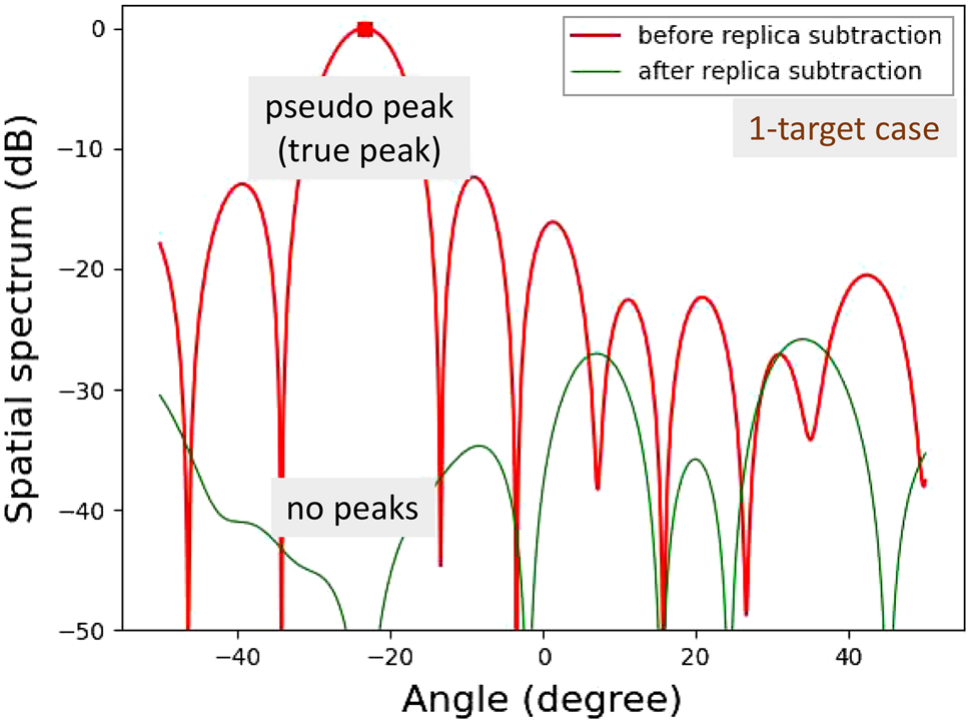
Experimental results for vertical angle estimations in a single-target case (3 m away).

**Figure 17:**
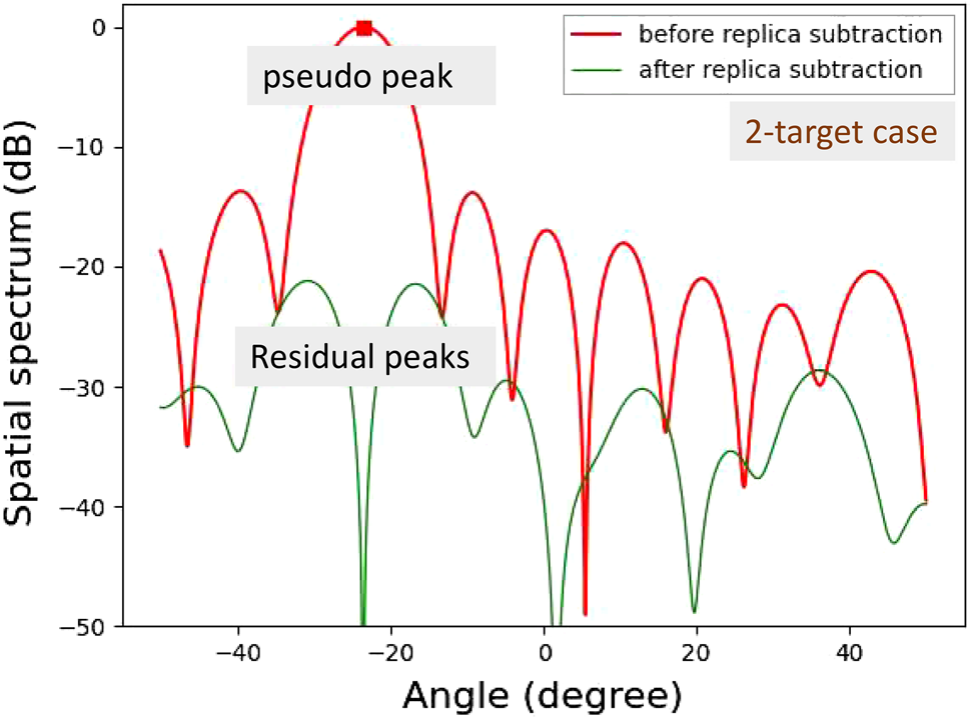
Experimental results for vertical angle estimations for a two-target case (3 m away with targets having the same radar cross-sectional area).

Figures 16 and 17 show experimentally obtained spatial spectra for a single incident wave and two incident waves, respectively.

For the single incident wave case shown in Fig. 16, a residual incident wave level around the peak angle is low enough; thus, the number of targets was successfully estimated to be one. The spatial spectrum around the incident angle was -196 dB. Note that the spatial spectrum outside of the incident angle region is higher because of the presence of noise and imperfect phase and amplitude calibrations for the RF circuits and antennas. This is the reason why the spatial spectrum after APPS does not become low enough contrary to that for the computationally obtained spatial spectrum shown in Fig. 5.

For the two incident waves shown in Fig. 17, the spatial spectrum before APPS shows a peak angle, and then a replica signal vector is generated for this angle. This replica signal vector was subsequently subtracted from the original receive signal vector. Higher residual incident wave levels in the spatial spectrum after APPS around the peak angle were observed, and, hence, this peak angle was a pseudo angle. Therefore, the number of incident waves was estimated to be two.

**Figure 18:**
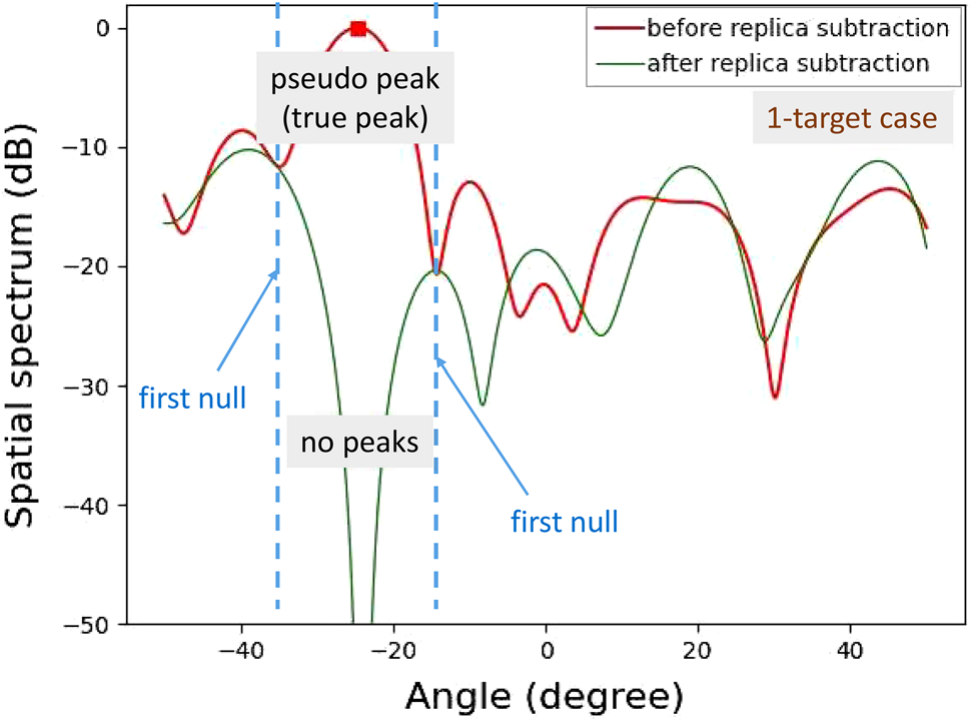
Experimental results for vertical angle estimations in a single-target case (20 m away).

**Figure 19:**
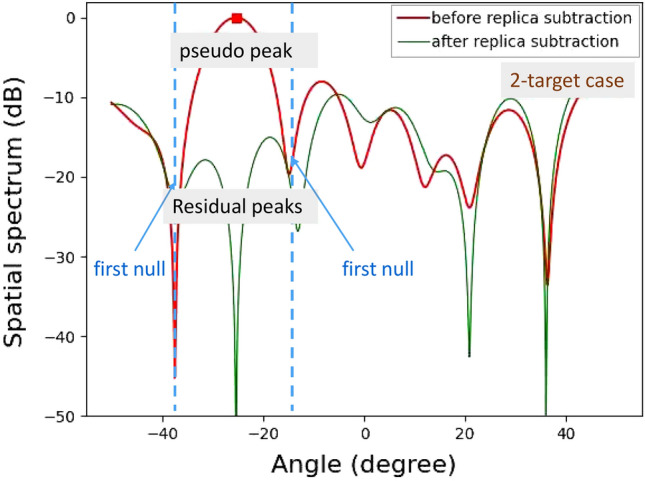
Experimental results for vertical angle estimations for a two-target case (20 m away with targets having the same radar cross-sectional area).

We also verify the performance of long-distance. Figures 18 and 19 experimentally obtained spatial spectra when one or two targets are placed 20 m away from the radar. The residual peaks for the two-target case shown in Fig. 19 are also obviously higher than that of the single-target case shown in Fig. 18. Since the spatial spectra have been normalized, the proposed method is less affected by the distance between radar and targets.

**Figure 20:**
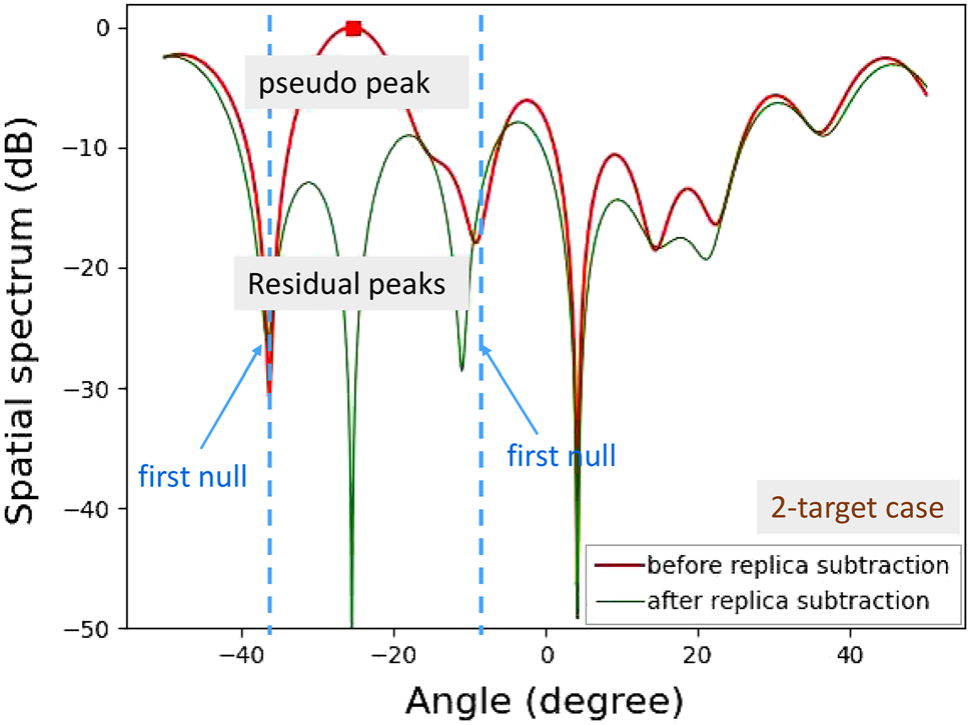
Experimental results for vertical angle estimations for a two-target case (20 m away with targets having different radar cross-sectional area).

Moreover, Fig. 20 shows the spatial spectrum when the two targets have different radar cross-sectional areas: 10 dBsm and 20 dBsm. Comparing Fig. 20 with Fig. 18 also finds obvious residual peaks for the two-target case with different radar cross-sectional areas.

**Figure 21:**
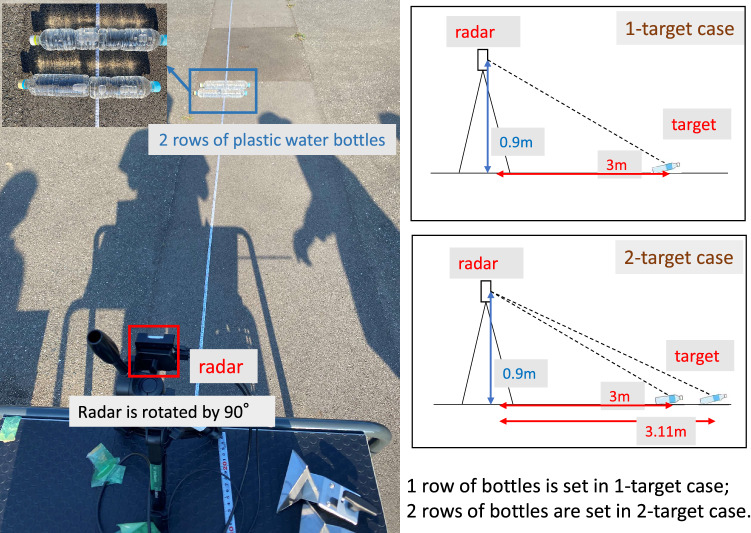
Demonstration environment for vertical angle estimations of on-road obstacles: plastic water bottles.

**Figure 22:**
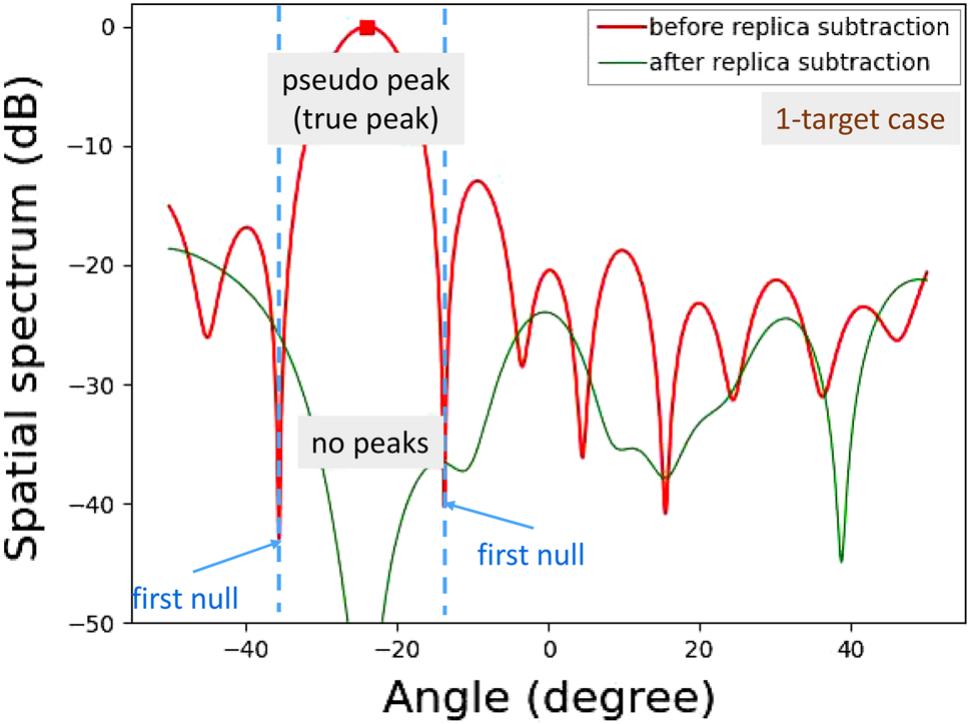
Demonstration results for vertical angle estimations of on-road obstacles: 1-target case.

**Figure 23:**
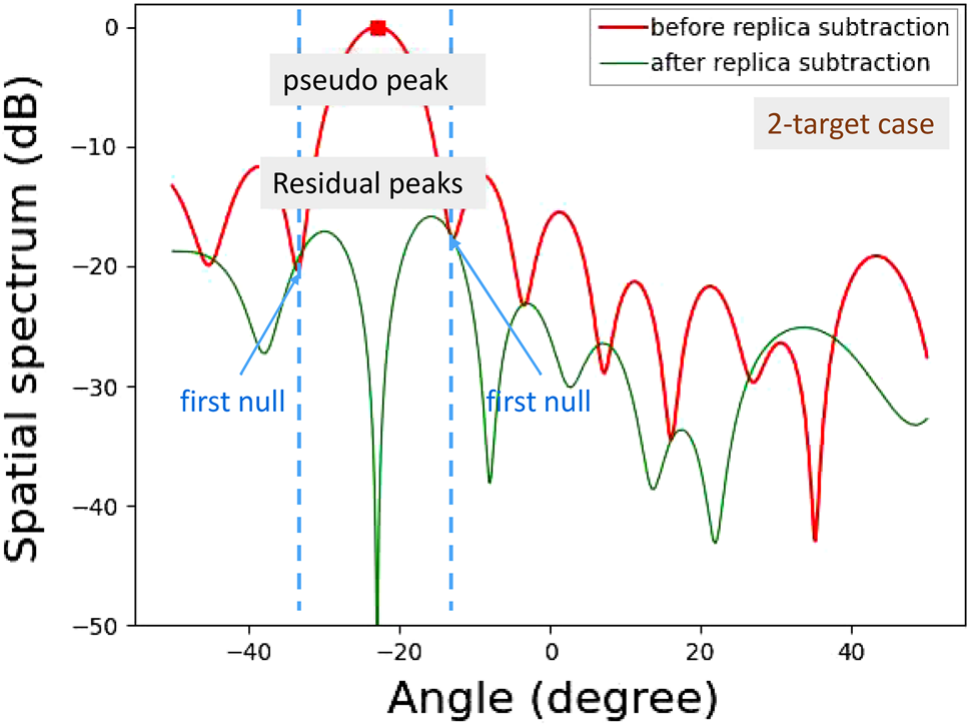
Demonstration results for vertical angle estimations of on-road obstacles: 2-target case.

For more practically validating the APPS processing, experiments for detecting plastic water bottles on an asphalt roadway were conducted. The plastic water bottles were placed on the asphalt roadway as on-road obstacles in Fig. 21. These bottles were filled with tap water. One row and two rows of bottles were used for 1-target and 2-target cases, respectively. For the 1-target case shown in Fig. 22, a residual incident wave level around the peak angle (between the first nulls in the spatial spectrum) is lower than -30 dB. For the 2-target case shown in Fig. 23, the residual incident wave levels around the peak angle in the spatial spectrum after replica subtraction by APPS were approximately -15 dB and two peaks were observed between the first nulls. Hence, the peak angles for the two targets case were pseudo. These results confirmed that the number of bottle rows was estimated.

### Performance analysis for angle estimation by APPS

The angle estimation performances were also experimentally evaluated, where the experimental environment is the same as that shown in Fig.15. The spacings between the two reflectors on the ground were changed for various angle differences.

**Figure 24:**
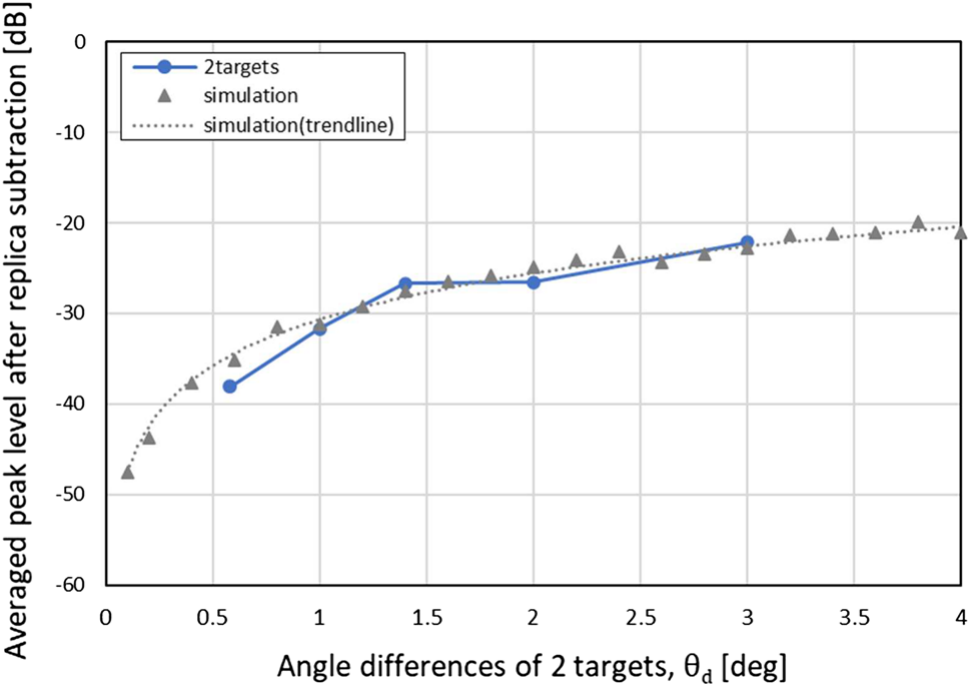
Experimentally obtained relationship between average residual levels and true angle differences. The obtained results are also compared with computationally obtained results.

Figure 24 shows the experimentally obtained relationship between average levels of residual incident waves and angle differences for the two targets. Several millimeter deviations were applied to one of the two reflectors to change the relative phase of the incident waves and to obtain an average residual level. The wavelength of 79 GHz radio waves is approximately 4 mm. In total, ten different relative phase realizations were used to obtain an average residual level. For the angle estimation, a reference curve was computationally obtained. As illustrated in Fig. 24, the experimentally obtained result is consistent with the simulation result, which validates the angle estimation performance of the APPS.

## Conclusion

This paper proposes a method to estimate the number of targets in a field of view, i.e., one target or two extremely close targets, along with their respective angles using a typical single-chip millimeter-wave radar IC. Two extremely closely located targets with an angle difference of approximately $0.5^\circ$ are experimentally resolved. A MIMO radar-based single-chip evaluation board with three TX and four RX antennas equipped with a uniform linear antenna array with an antenna spacing of approximately half a wavelength is used in the experiments. In the antenna element space, the proposed method subtracts a replica signal vector for a pseudo peak, and then the residual incident wave components observed in the spatial spectra are used to estimate the number of targets and their angles.

## Data Availability

The datasets used and/or analysed during the current study available from the corresponding author on reasonable request.
